# Serum MicroRNAs as Potential Biomarkers of Primary Biliary Cirrhosis

**DOI:** 10.1371/journal.pone.0111424

**Published:** 2014-10-27

**Authors:** Youwen Tan, Tengli Pan, Yun Ye, Guohong Ge, Li Chen, Danfeng Wen, Shengqiang Zou

**Affiliations:** 1 Department of Hepatosis, The Third Hospital of Zhenjiang Affiliated Jiangsu University, Zhenjiang, China; 2 Department of Infection, The People’s Hospital of Bozhou, Bozhou, China; Emory University School of Medicine, United States of America

## Abstract

**Background:**

Circulating microRNAs (miRNAs), which are extremely stable and protected from RNAse-mediated degradation in body fluids, have emerged as candidate biomarkers for many diseases. The present study aimed to identify a serum microRNA (miRNA) expression profile that could serve as a novel diagnostic biomarker for primary biliary cirrhosis (PBC).

**Methods:**

Serum miRNA expression was investigated using four cohorts comprising 380 participants (healthy controls and patients with PBC) recruited between August 2010 and June 2013. miRNA expression was initially analyzed by Illumina sequencing using serum samples pooled from 3 patients and 3 controls. Quantitative reverse transcriptase polymerase chain reaction (qRT-PCR) was then used to evaluate the expression of selected miRNAs in a screening set (n = 40). A logistic regression model was then constructed using a training cohort (n = 192) and validated using another cohort (n = 142). The area under the receiver operating characteristic curve (AUC) was used to evaluate diagnostic accuracy.

**Results:**

We identified a miRNA panel (hsa-miR-122-5p, hsa-miR-141-3p, and hsa-miR-26b-5p) with a high diagnostic accuracy for PBC (AUC = 0.905, 95% confidence interval (CI) = 0.857 to 0.953; sensitivity = 80.5%, specificity = 88.3%). There was a significant difference between AUC values of the miRNA panel and those of alkaline phosphatase (ALP) (AUC = 0.537, difference between areas = 0.314, 95% CI = 0.195 to 0.434, *P*<0.001), and those of antinuclear antibody (ANA) (AUC = 0.739, difference between areas = 0.112, 95% CI = 0.012 to 0.213, *P* = 0.0282).

**Conclusion:**

We identified a serum microRNA panel with considerable clinical value in PBC diagnosis. The results indicate that the miRNA panel is a more sensitive and specific biomarker for PBC than ALP and ANA.

## Introduction

Primary biliary cirrhosis (PBC) is a female predominant, progressive autoimmune disease characterized by immune-mediated destruction of the intrahepatic bile ducts. PBC characteristic serologic hallmark is the anti-mitochondrial antibody (AMA), a highly disease-specific autoantibody found in 90–95% of patients and less than 1% of normal controls [1]. AMA directed against the E2 subunit of the pyruvate dehydrogenase enzyme complex located in the inner mitochondrial membrane is the most important reference standar [2]. However, fewer than 5% of patients with PBC are AMA-negativ [3]. PBC diagnosis is established based on the following criteria: (1) biochemical evidence of cholestasis, (2) the presence of AMA, and (3) histopathologic evidence of nonsuppurative cholangitis and destruction of the interlobular bile duct [4]. Though diagnostic criteria have been determined, the progression to biochemically and clinically apparent disease is unpredictable. Many patients are diagnosed at an early stage of disease and respond well to medical therapy, while some patients will require liver transplantatio [5]. To revolutionize the diagnosis, treatment, and prognosis of PBC, new biomarkers should be identified. MicroRNAs (miRNAs) are emerging as highly tissue-specific biomarkers with potential clinical applicabilit [6].

MiRNAs are an emerging class of highly conserved, non-coding small RNAs that regulate gene expression at the post-transcriptional level. It is now clear that miRNAs can potentially regulate every aspect of cellular activity, including differentiation and development, metabolism, proliferation, apoptotic cell death, viral infection, and tumorigenesis [7]. Recent studies provide clear evidence that miRNAs are abundant in the liver and modulate a diverse spectrum of liver functions [8]. Deregulation of miRNA expression may be a key pathogenic factor in many liver diseases, including viral hepatitis, hepatocellular cancer, and polycystic liver disease. A clearer understanding of the mechanisms involved in miRNA deregulation would offer new diagnostic and therapeutic strategies to treat liver diseases. Circulating miRNAs, which are extremely stable and protected from RNAse-mediated degradation in body fluids, have emerged as candidate biomarkers for many diseases [9], [10], [11]. The use of miRNAs as noninvasive biomarkers is of particular interest in liver diseases [12], .

Since the initial study by Qin et al. [15], 17miRNAs have been identified to be differentially expressed in PBMCs from patients with PBC. Ninomiya et al. [16] also found that the down-regulation of hsa-miR-505-3p and miR-197-3p expression can serve as clinical biomarkers of PBC.

Our study investigated miRNA expression profiles with independent validation in a large cohort of participants, in order to identify a panel of miRNAs for the diagnosis of PBC. The cohort included healthy individuals and patients with PBC.

## Materials and Methods

### Ethics statement

The study was approved by the Medical Ethics Committee of The Third Hospital Affiliated to Jiangsu University (No. 201002) and written informed consent was obtained from each patient prior to participation. The study was conducted in accordance with the Declaration of Helsinki.

### Study design, patients, and healthy controls

**Figure 1:**
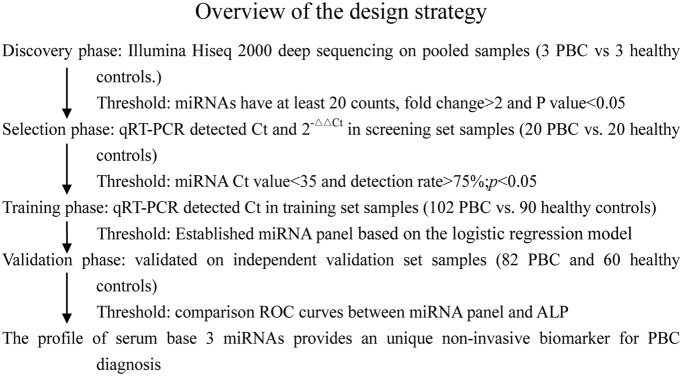
A flow chart of the experimental design.

A multistage, case-control study was designed to identify a serum miRNA profile as a surrogate marker for PBC (Fig. 1). A total of 207 patients with PBC and 173 healthy controls were enrolled in our study. In the biomarker discovery stage, 6 serum samples pooled from 3 healthy control donors and 3 patients with PBC were subjected to Illumina Hiseq 2000 deep sequencing to identify miRNAs that were significantly differentially expressed. In the biomarker selection stage, the expression of different miRNAs was validated by quantitative reverse transcriptase polymerase chain reaction (qRT-PCR) in samples from 20 patients with PBC and 20 healthy controls. Subsequently, samples from 102 patients with PBC and 90 healthy controls were used in the training set. Sequential validation was performed using a hydrolysis probe-based qRT-PCR assay to refine the number of serum miRNAs as a PBC signature, while samples from an additional 82 patients with PBC and 60 healthy controls serum samples were used in an independent validation set. All patients were diagnosed with PBC between August 2010 and June 2013 (from The Third Hospital of Zhejiang Affiliated Jiangsu University) and blood samples were collected prior to any therapeutic procedure. Patients who had abnormal liver enzyme values regardless of histologic stage were receiving ursodeoxycholic acid (UDCA) in a dose of 13–15 mg/kg/day orally. PBC diagnosis can be established when two of the following three criteria are met: (1) biochemical evidence of cholestasis based mainly on alkaline phosphatase (ALP) elevation, (2) presence of AMA, and (3) histologic evidence of nonsuppurative destructive cholangitis and destruction of interlobularbile duct [4]. The clinical phases were divided into four phases: preclinical, asymptomatic, symptomatic, and liver insufficienc [17]. Patients with other disorders such as drug-induced liver disease, alcoholic liver disease, viral hepatitis, schistosomiasis, autoimmune hepatitis, sclerosing cholangitis, α1-antitrypsin deficiency, hemochromatosis, Wilson’s disease, and biliary obstruction were excluded. Healthy control subjects were recruited from a large pool of individuals seeking a routine health check-up at the Healthy Physical Examination Centre of The Third Hospital of Zhejiang Affiliated Jiangsu University. The healthy controls were also required to have normal ALT level (ALT<40 IU/mL) and no history of coronary heart disease, hypertension, valvular disease, any arrhythmia, or systemic disease for inclusion in the study. The controls and patients were matched based on age, gender, and ethnicity.

### RNA isolation and library preparation

About 5 mL of venous blood was collected from each participant. The whole blood was separated into serum and cellular fractions by centrifugation at 4,000 rpm for 10 min, followed by centrifugation at 13,000 rpm for 5 min for complete removal of cell debris. The supernatant serum was stored at –80°C until analysis. Total RNA was isolated using LCS TRK1001 miRNeasy kit (LC Sciences, Hangzhou, China). The libraries were constructed from total RNA using the Illumina Truseq Small RNA Sample Preparation Kit (Illumina, San Diego, CA, USA) according to the manufacturer’s protocol. Briefly, RNA 3′ (P-UCGUAUGCCGUCUUCUGCUUG-UidT) and 5′ (GUUCAGAGUU CUACAGUCCGACGAUC) adapters were ligated to target miRNAs in two separate steps. Reverse transcription reaction was applied to the ligation products to create single stranded cDNA. The cDNA was amplified by PCR using a common primer and a primer containing the index sequence (CAAGCAGAAGACGGCATACGA). The quantity and purity of total RNAs were monitored using a NanoDrop ND-1000 spectrophotometer (NanoDrop Inc., Wilmington, DE, USA) at a 260/280 ratio >2.0. The integrity of total RNAs was analyzed using an Agilent 2100 Bioanalyzer system and RNA 6000 Nano LabChip Kit (Agilent Tech, Santa Clara, CA, USA) with RNA integrity number >8.0. Finally, Illumina sequencing technology was employed to sequence these prepared samples.

### Illumina sequencing and data analysis

The raw sequences were processed using the Illumina pipeline program. After masking of the adaptor sequences and removal of contaminated reads, the clean reads were filtered for miRNA prediction with the software package ACGT101-miR-v4.2 (LC Sciences, Houston, Texas, USA) and subsequently analyzed according to report [18]. Secondary structure prediction of individual miRNAs was performed by Mfold software (Version 2.38; http://mfold.rna.albany.edu/?q=mfold/RNA-Folding-Form) using the default folding conditions. The raw data were reduced to cleaned sequences by removal of the following sequences: (1) 3ADT&length filter: reads were removed due to 3ADT not being found and reads with length <18 and >26 were removed; (2) Junk reads: Junk: ≥2N, ≥7A, ≥8C, ≥6G, ≥7T, ≥10Dimer, ≥6Trimer, or ≥5Tetramer; (3) Rfam: Collection of many common non-coding RNA families except miRNAs (http://rfam.janelia.org); (4) Repeats: Prototypic sequences representing repetitive DNA from different eukaryotic species (http://www.girinst.org/repbase); (5) Notes: There was an overlap in mapping reads with mRNA, rRNA, tRNA, snRNA, snoRNA, and repeats; (6) mRNA Database: (http://www.ncbi.nlm.nih.gov/). The clean sequence reads were mapped with miRBase 20.0, allowing a mismatch of one or two nucleotide bases. The computational pipeline employed for data handling is reported in the flowchart of the study procedure (Figure S1). All data were transformed to log base 2. Differences between the samples were calculated using chi-square and fisher’s exact test. Only miRNAs with fold difference >2.0 and *P*<0.05 were considered statistically significant.

### qRT-PCR validation study and data analysis

Relative quantification of miRNAs by qRT-PCR (300 µL of serum from each participant) was performed with SYBR Premix Ex Taq (TaKaLa) according to the manufacturer’s instructions using a Rotor-Gene 3000 Real-time PCR machine (Corbett Life Science, Sydney, Australia). The RT primers and realtime PCR primers were designed as previously describe [19]. Briefly, 1 µg of total RNA was reverse transcribed under the following conditions: 16°C for 15 min, 42°C for 60 min, and 85°C for 5 min. The PCR total volume was 20 µL and included 1 µL of RT product and 1 µL EvaGreen dye (Biotium, Hayward, CA, USA). The PCR reaction conditions were as follows: 95°C for 5 min followed by 40 cycles of 95°C for 15 s and 60°C for 1 min using an ABI PRISM 7300 thermal cycler. All reactions were run in triplicate. The threshold cycle (Ct) is defined as the fractional cycle number at which the fluorescence passes the fixed threshold. According to previous studies, miRNA-24 is consistently expressed in human serum [20], [21]. Moreover, our previous experience is that miRNA-24 expression is stable and that its expression level can serve as an internal control in serum miRNA relative quantitative analysis. The specificity of each PCR product was validated by melting curve analysis at the end of PCR. All samples were analyzed in triplicate and the cycle threshold value was defined as the number of cycles required for the fluorescent signal to reach the threshold. miRNA relative expression levels in serum were calculated using the formula 2^−ΔΔCt^ where ΔΔCt = [Ct (target, test)−Ct (ref, test)]−[Ct (target, calibrator)−Ct (ref, calibrator)] [22]. All primers used were obtained from Invitrogen (Shanghai, China).

### Statistical analysis

All Illumina sequencing data were transformed to log base 2. Differences between the samples were calculated using chi-square and fisher’s exact test. Only miRNAs with fold difference >2.0 and *P*<0.05 were considered statistically significant. Data are presented as median ± SD. Demographic and clinical characteristics of patients with PBC and healthy controls were analyzed using the Statistical Package for the Social Sciences (SPSS) version 21.0 software (SPSS Inc, Chicago, IL, USA). For miRNA 2^−ΔΔCt^ values obtained by qRT-PCR, Mann-Whitney unpaired test was used to compare the results from patients with PBC to that of the healthy controls. A stepwise logistic regression model was used to select diagnostic miRNA markers based on the training dataset. The predicted probability of being diagnosed with PBC was used as a surrogate marker to construct the receiver operating characteristic (ROC) curve. Area under the ROC curve (AUC) was used as an accuracy index for evaluating the diagnostic performance of the selected miRNA panel. The ROC and regression analysis was performed using the software 21MedCalc (Version 10.4.7.0; MedCalc, Mariakerke, Belgium). All *P* values were two-sided.

## Results

### Description and clinical features of the patients with PBC

All 207 patients enrolled in the present study were clinically diagnosed with PBC. As shown in Table 1, there was no significant difference in the distribution of smoking, alcohol consumption, age, and gender between patients with PBC and normal subjects. However, the total bilirubin (TBIL), ALT, AST, and ALP of patients with PBC were significantly different from those of the normal controls.

**Table 1:** Demographic and clinical features of the PBC patients and healthy controls in the screening set, training set and the validation set.

	screening set					training set					validation set				
Variables	PBCs (n = 20)		Controls (n = 20)		*p*-value	PBCs (n = 102)		Controls (n = 90)		*p*-value	PBCs (n = 82)		Controls (n = 60)		*p*-value
	N0.	%	N0.	%		N0.	%	N0.	%		N0.	%	N0.	%	
Average age (years)	46.13±11.01		45.88±10.17		*p* = 0.626^a^	48.21±10.81		48.11±9.22		*p* = 0.661^a^	48.20±8.84		48.63±10.02		*p* = 0.063^a^
Sex															
Male	2	10	3	15	*p* = 0.633^b^	6	5.9	5	5.6	*p* = 0.923^b^	6	7.4	4	6.7	*p* = 0.865^b^
Female	18	90	17	85		96	94.1	85	94.4		75	92.6	56	93.3	
Smoking status															
Ever	1	5	2	10	*p* = 0.833^b^	2	2	3	3.3	*p* = 0.837^b^	2	2.4	2	3.3	*p* = 0.933^b^
Current	3	15	3	15		7	6.9	6	6.7		5	6	4	6.7	
Never	16	80	15	75		93	91.1	81	90		76	91.6	54	90	
^ 1^BMI	24.75±3.24		24.37±3.63		*P* = 0.564	25.31±3.54		25.12±3.09		*P* = 0.886	24.87±3.44		24.64±4.15		*P* = 0.765
Alcohol consumption															
Occasional^2^	12	60	11	55	*p* = 0.749^b^	54	52.9	38	42.2	*p* = 0.138^b^	53	63.9	37	61.7	*p* = 0.789^b^
Never	8	40	9	45		48	47.1	52	57.8		30	36.1	23	38.3	
AMA^3^															
Positive	20	100	0	0	*p* = 0.000^b^	96	94.1	0	0	*p* = 0.000^b^	80	97.6	0	0	*p* = 0.000^b^
Negative	0	0	20	100		6	5.9	90	100		2	2.4	60	100	
ANA^4^	30.76±38.1		5.415±1.912		*p* = 0.008^a^	34.53±34.27		5.353±1.353		*p* = 0.005^a^	33.252±40.273		5.374±2.643		*p* = 0.011^a^
TBIL (Umol/L)	19.51±15.12		9.57±3.12		*p* = 0.009^a^	21.94±16.74		10.91±3.42		*p* = 0.000^a^	26.54±10.14		11.78±2.85		*p* = 0.000^a^
ALT (U/L)	155.1±278.85		27.35±7.48		*p* = 0.048^a^	115.26±176.22		30.96±6.27		*p* = 0.000^a^	116.69±142.84		32.62±5.39		*p* = 0.000^a^
AST (U/L)	147.75±248.95		27.5±4.81		*p* = 0.037^a^	111.43±186.57		30.13±6.26		*p* = 0.000^a^	114.3±125.93		30.68±6.6		*p* = 0.000^a^
ALP (U/L)	160.70±190.51		76.65±43.72		*p* = 0.044^a^	138.86±146.86		70.66±21.82		*p* = 0.000^a^	154.7±157.61		80.78±42.8		*p* = 0.003^a^
Clinical phases															
Preclinical	4	20				8	7.8				8	9.8			*p* = 0.635^b^
Asymptomatic	4	20				21	20.6				15	18.3			
Symptomatic	6	30				32	31.4				21	25.6			
Liver insufficiency	6	30				41	40.2				38	46.3			

1 BMI, Body mass index,

2 the ethanol intake per week was less than 140 g in men (70 g in women) in the past 12 months.

3 antimitochondrial antibody,

4 antinuclear antibody;

a Independent samples-t test.

b Pearson Chi-Square.

### Global analysis of miRNAs by deep sequencing

**Figure 2:**
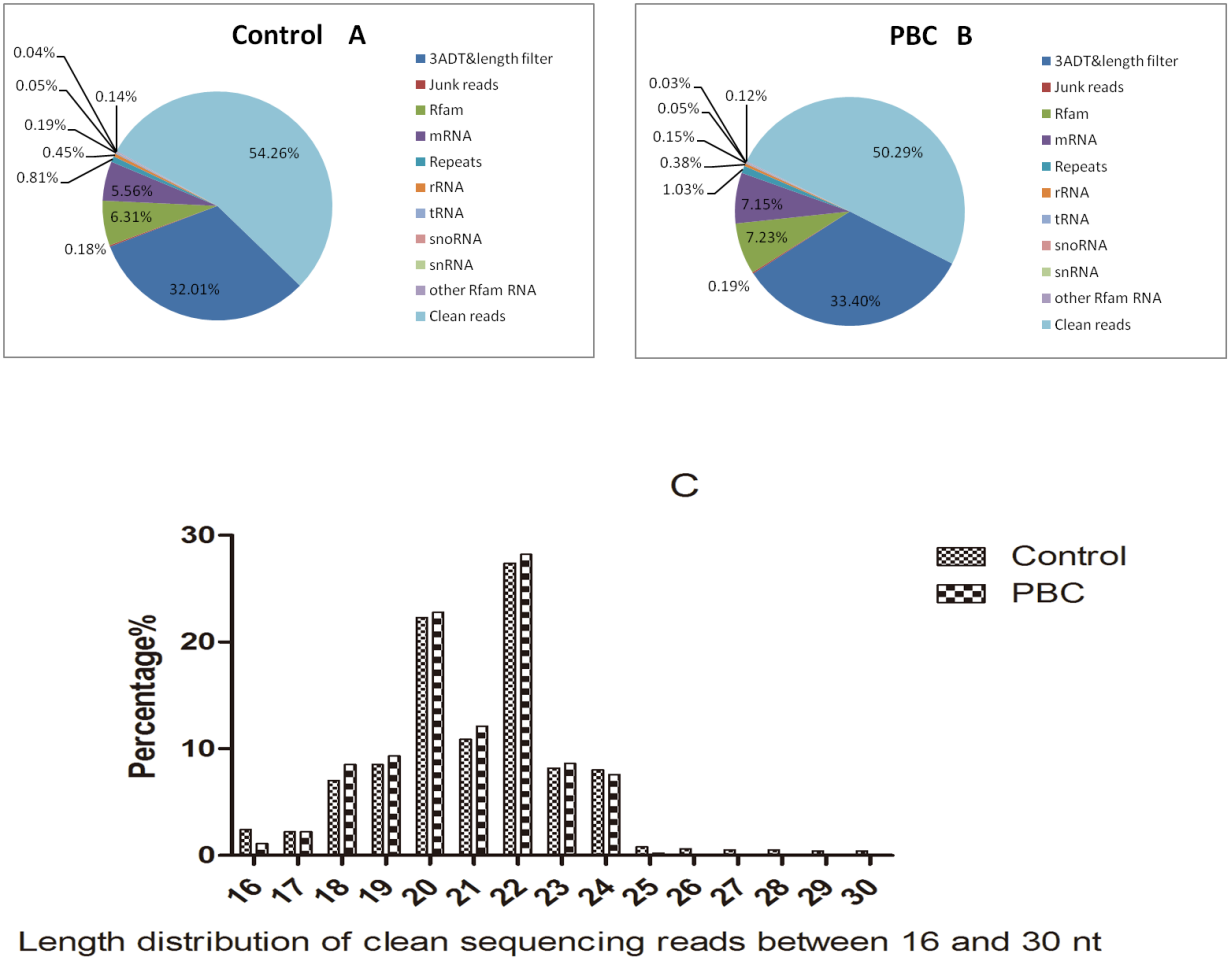
Sequenced reads and distribution of reads. The Illumina Hiseq 2000 sequencing of the small RNA library from the serum of healthy controls and PBC patients produced 8,580,434 and 9,371,001 raw-reads, respectively. After extensive preprocessing and quality control, these raw reads were reduced to 659,447 and 482,263 clean reads, indicating 54.26% and 50.29% of sequenced reads, respectively (Fig. 2A, 2B). The distribution of all reads from 16 to 30 nt is presented in Fig. 2C.

The Illumina Hiseq 2000 sequencing of the small RNA library from the serum of healthy controls and patients with PBC produced 8,580,434 and 9,371,001 raw-reads, respectively. After extensive preprocessing and quality control, these raw reads were reduced to 659,447 and 482,263 clean reads, indicating 54.26% and 50.29% of sequenced reads, respectively (Fig. 2A, 2B, Table S1). The distribution of all reads from 16 to 30 nt is presented in Fig. 2C. In our study, we found that the miRNA length was mainly 18 and 24 nt. The clean reads were then mapped to the human miRNA database v20.0 (ftp://mirbase.org/pub/mirbase/CURRENT/), pre-miRNA (mirs) database v20.0 (ftp://mirbase.org/pub/mirbase/CURRENT/), and genome database (ftp.ncbi.nih.gov/genomes/H sapiens/Assembled chromosomes/seq/). A total of 1,768 unique reads could be mapped to human miRNAs or pre-miRNAs in miRbase and the pre-miRNAs could be further mapped to the human genome and expressed sequence tag.

### Analysis of differentially expressed miRNAs

The differential expression of miRNA count data was normalized and the number of individual miRNA reads was standardized by the total numbers of 1,000,000 reads in each sample. Comparing the PBC and healthy control groups, 126 miRNAs presented significant differential expression levels. Among them, 17 miRNAs were upregulated (fold change >2-fold, *P*<0.05) in the control group,1 was downregulated (fold change >2-fold, *P*<0.05), as shown in Table 2.

**Table 2:** 17 differentially expression miRNAs between control and PBC.

no.	miR_name	fold change (log)	fold change	up/down	miR_seq
1	hsa-miR-122-5p	3.13	8.76	up	TGGAGTGTGACAATGGTGTTT
2	hsa-miR-34a-5p	2.86	7.26	up	UGGCAGUGUCUUAGCUGGUUGU
3	hsa-miR-200a-3p_R−1	2.32	4.98	up	TAACACTGTCTGGTAACGATG
4	hsa-miR-141-3p	2.27	4.84	up	UAACACUGUCUGGUAAAGAUGG
5	hsa-miR-215–5p_R−1	2.10	4.27	up	ATGACCTATGAATTGACAGA
6	hsa-miR-21-3p	1.60	3.03	up	CAACACCAGTCGATGGGCTGT
7	hsa-miR-320c_R−4	1.51	2.86	up	AAAAGCTGGGTTGAGA
8	hsa-miR-21–5p	1.36	2.56	up	TAGCTTATCAGACTGATGTTGA
9	hsa-miR-193a-5p	1.33	2.51	up	TGGGTCTTTGCGGGCGAGATGA
10	hsa-miR-26b-5p	−1.32	2.49	down	TTCAAGTAATTCAGGATAGGT
11	hsa-miR-194-5p	1.31	2.48	up	UGUAACAGCAACUCCAUGUGGA
12	hsa-miR-27b-3p	1.29	2.44	up	TTCACAGTGGCTAAGTTCTG
13	hsa-miR-320a	1.27	2.41	up	AAAAGCTGGGTTGAGAGGGCGA
14	hsa-miR-210-3p_R−2	1.27	2.41	up	CTGTGCGTGTGACAGCGGCT
15	hsa-miR-22-3p	1.06	2.09	up	AAGCTGCCAGTTGAAGAACTGT
16	hsa-miR-1246_L−2R+1	1.05	2.07	up	TGGATTTTTGGAGCAGGG
17	hsa-miR-152-3p	1.01	2.02	up	TCAGTGCATGACAGAACTTGG

### Differential Expression Profile of Five Selected miRNAs

The expression of 17 candidatemiRNAs that were selected from the previous step was confirmed by qRT-PCR in an independent cohort of 40 serum samples. Threshold levels were found to be as follows: miRNA Ct <35 and detection rate >75%. We determined the 2^−ΔΔCt^ of 17 candidate miRNAs in the two groups, Mann-Whitney unpaired test was used to compare miRNA expression between patients with PBC and controls. Five of the 17 miRNAs presented significantly different expression levels between the PBC and control group, as shown in Table 3. These were hsa-miR-122-5p, hsa-miR-34a-5p, hsa-miR-141-3p, hsa-miR-26b-5p, and hsa-miR-27b-3p.

**Table 3:** Expression profiles of 17 candidate miRNA on qRT-PCR in screening set.

no.	miR_name	p value	fold change
1	**hsa-miR-122-5p**	0.0000	9.73
2	**hsa-miR-34a-5p**	0.0000	5.76
3	hsa-miR-200a-3p_R−1	0.6020	4.36
4	**hsa-miR-141-3p**	0.0000	3.45
5	hsa-miR-215-5p_R−1	0.6020	3.41
6	hsa-miR-21-3p	0.1080	2.81
7	hsa-miR-320c_R−4	ND	ND
8	hsa-miR-21-5p	0.2890	2.18
9	hsa-miR-193a-5p	0.2890	2.23
10	**hsa-miR-26b-5p**	0.0060	0.64
11	hsa-miR-194-5p	1.0000	1.14
12	**hsa-miR-27b-3p**	0.0060	3.12
13	hsa-miR-320a	ND	ND
14	hsa-miR-210-3p_R−2	ND	ND
15	hsa-miR-22-3p	0.2890	1.96
16	hsa-miR-1246_L−2R+1	ND	ND
17	hsa-miR-152-3p	0.1080	2.02

ND: not determined, miRNA Ct value>35 and detection rate<75%.

### miRNA expression profile in patients with PBC and healthy control in the training data set

**Figure 3:**
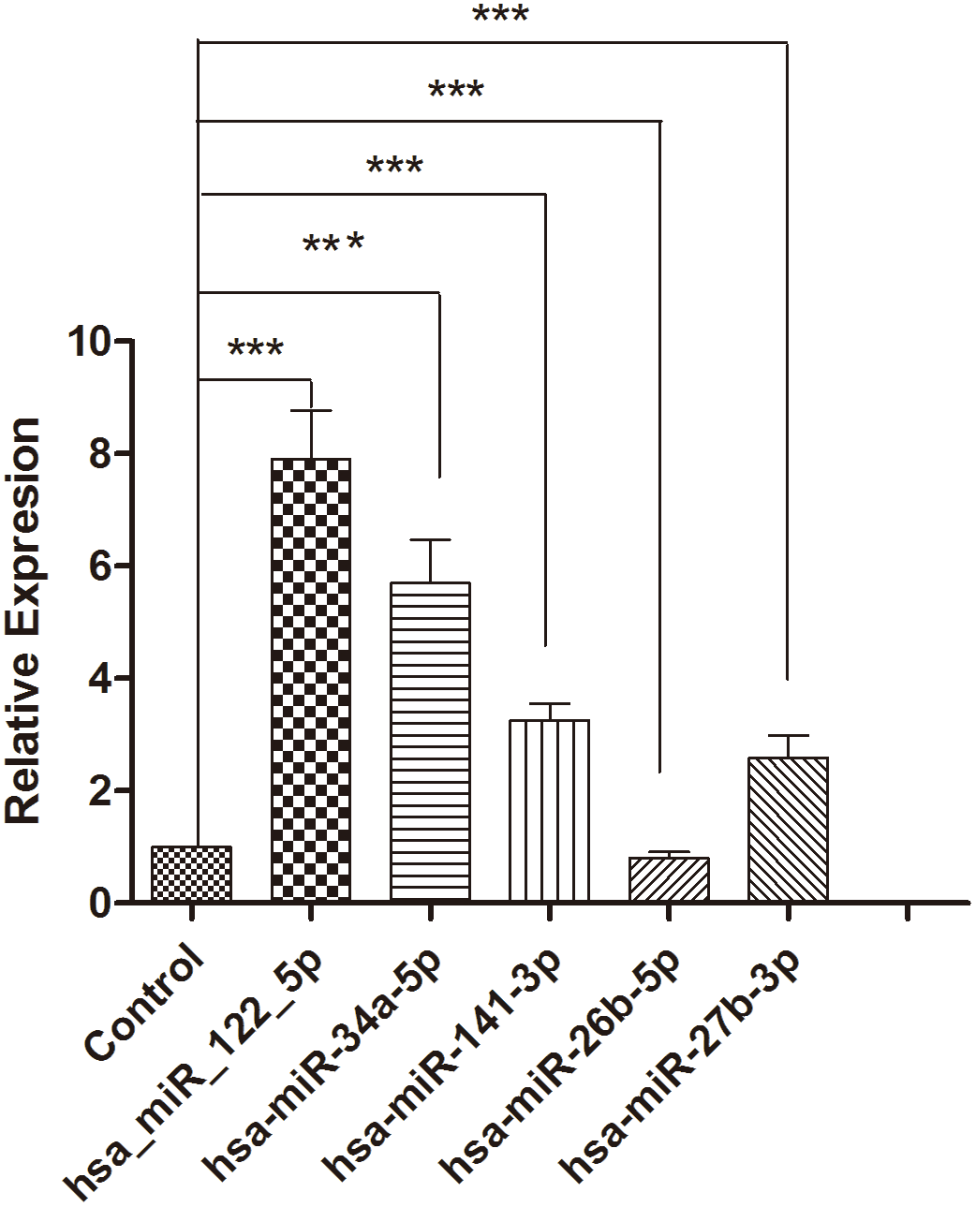
Relative expression of miRNAs between healthy controls and patients with PBC. Relative expression of 5 candidate miRNAs between controls and patients with PBC in the training set.

**Figure 4:**
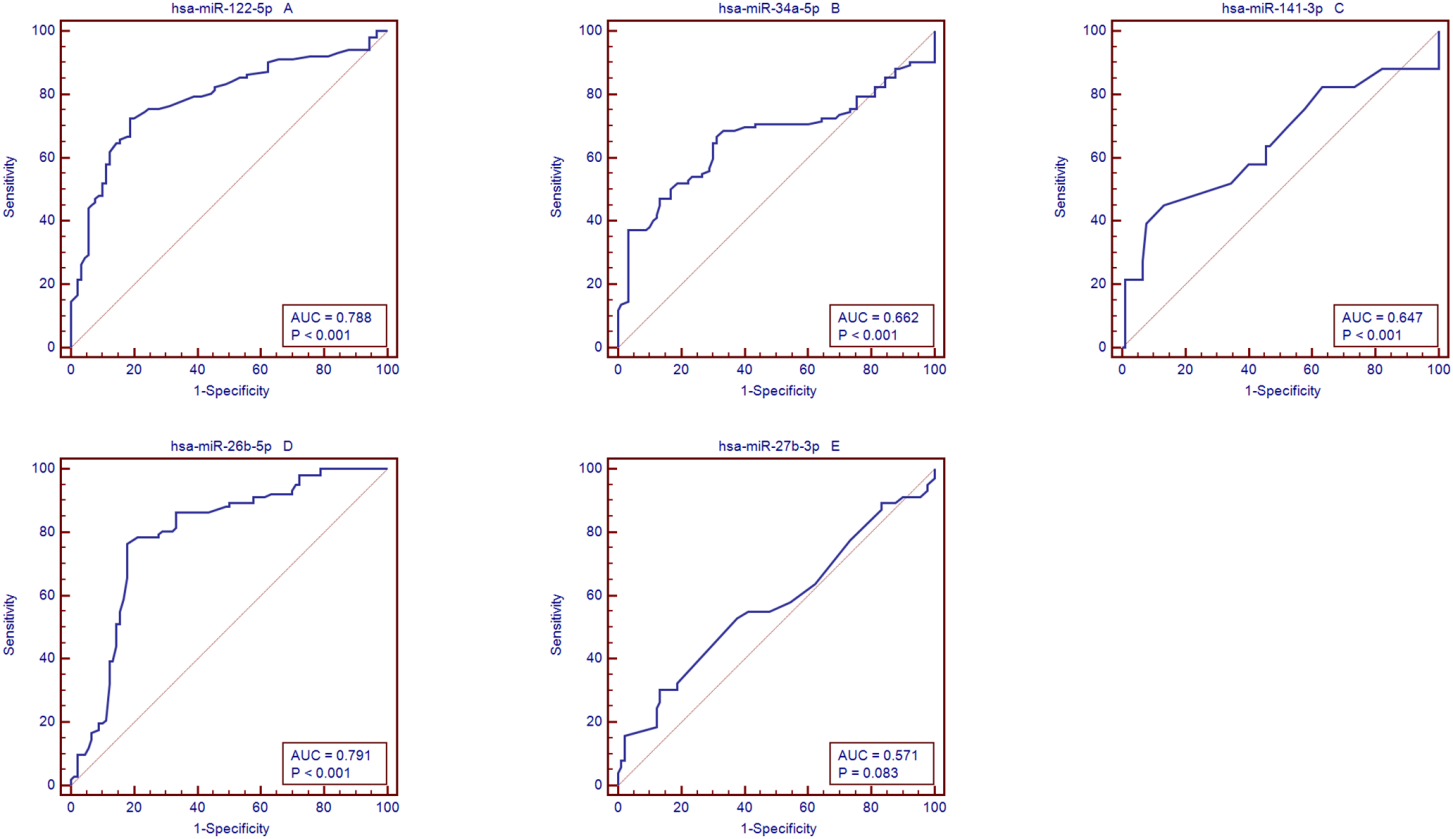
AUC of miRNAs between controls and patients with PBC. Area under the curve (AUC) of miRNAs. A: miRNA-122; B: hsa-miR-34a-5p; C: hsa-miR-141-3p; D: hsa-miR-26b-5p and E: hsa-miR-27b-3p.

qRT-PCR assay was used to confirm the expression of 5 candidate miRNAs that were selected from the previous step. In the training set, samples from 102 patients with PBC and 90 controls were examined by qRT-PCR. This phase generated a list of 5 miRNAs that presented a significant differential expression pattern (Fig. 3, Table 4), hsa-miR-122-5p, hsa-miR-34a-5p, hsa-miR-141-3p, hsa-miR-26b-5p, and hsa-miR-27b-3p. The diagnostic accuracy of these miRNAs, as measured by AUC, was 0.788, 0.662, 0.647, 0.791, and 0.571, respectively (Table S2, Fig. 4A–E).

**Table 4:** Expression profiles of 5 candidate miRNA on qRT-PCR in training set.

no.	miR_name	p value	fold change
1	hsa-miR-122-5p	0.0000	9.79
2	hsa-miR-34a-5p	0.0000	5.69
3	hsa-miR-141-3p	0.0000	3.25
4	hsa-miR-26b-5p	0.0030	0.78
5	hsa-miR-27b-3p	0.0138	2.54

### Establishing the predictive miRNA panel

**Figure 5:**
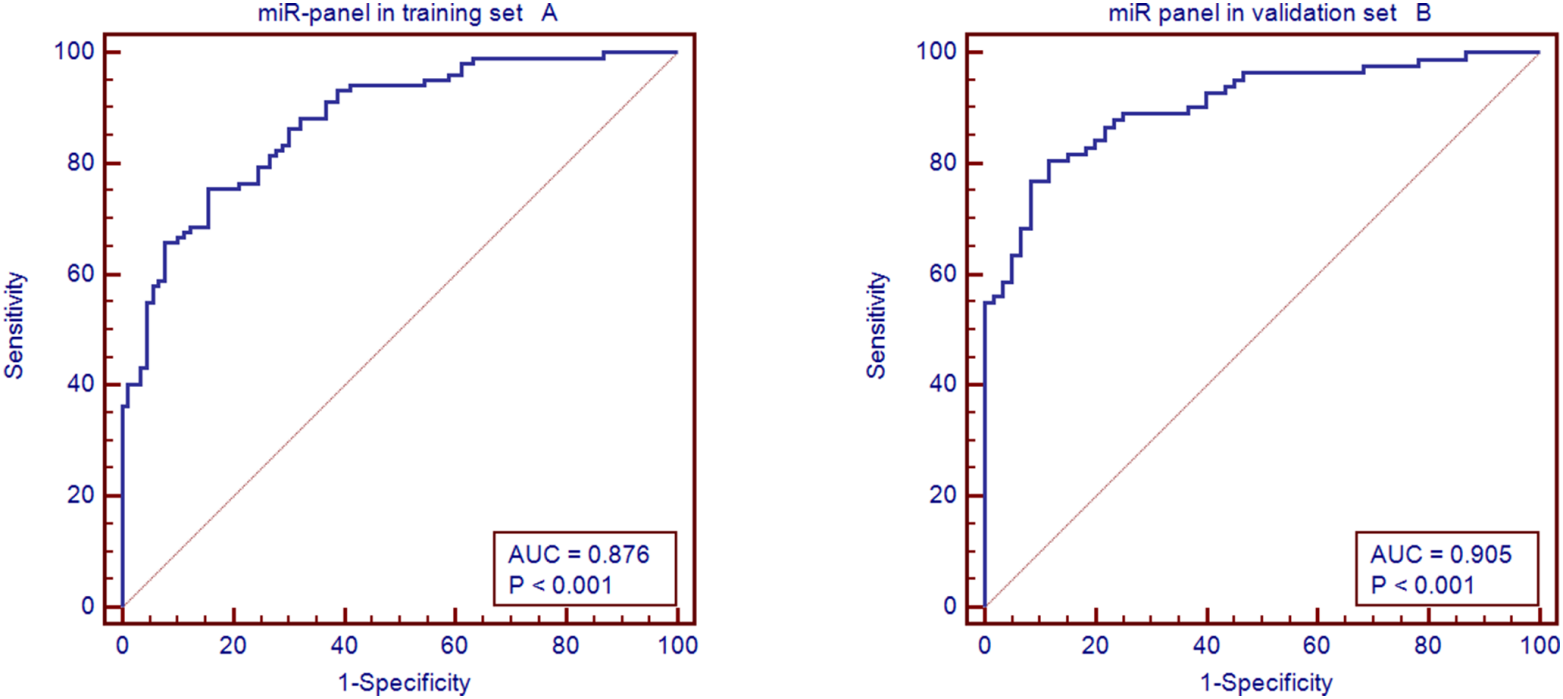
AUC of miRNA panel in the training set and validation set. A: AUC for the miRNA panel in the training set and B: AUC of the miRNA panel in the validation set.

A stepwise logistic regression model to estimate the risk of being diagnosed with PBC was applied to the training set (192 serum samples). Three of the five miRNAs turned out to be significant predictors (Threshold: Enter variable, if *P*<0.05;remove variable, if *P*>0.1,). The predicted probability of being diagnosed with PBC was determined from the three miRNA panel logit model (Table S3). LogitP = 10.2834−0.74034miR122−0.3616miR141+0.65338miR26b was used to construct the ROC curve. The diagnostic performance for the established miRNA panel was evaluated using ROC analysis. The miRNA panel AUC was 0.876 (95% CI = 0.829 to 0.924; sensitivity = 75.5%, specificity = 74.4%, Fig. 5A).

### Validating the miRNA panel

The parameters estimated from the training data set were used to predict the probability of being diagnosed with PBC using the independent validation set (142 serum samples). Similarly, the predicted probability was used to construct the ROC curve. The AUC of the miRNA panel was 0.905 (95% CI = 0.857 to 0.953; sensitivity = 80.5%, specificity = 88.3%, Fig. 5B).

**Figure 6:**
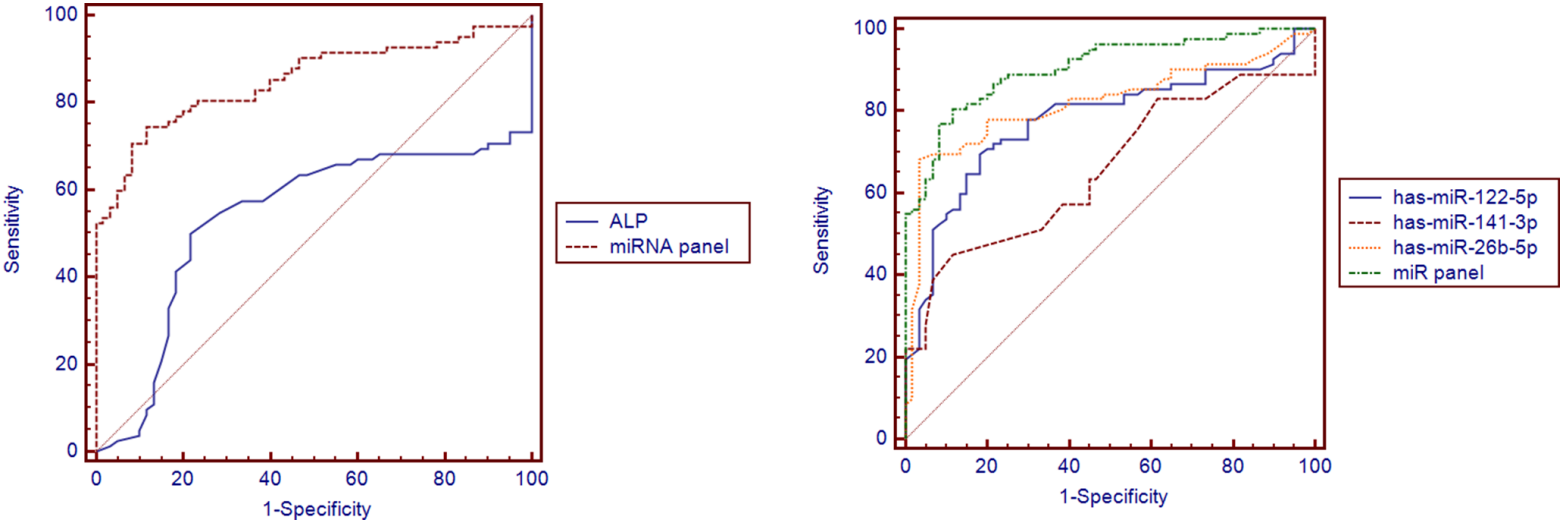
Comparison curves of ROC. A: Comparison curves of ROC between ALP and miRNA panel in the validation set; B: Comparison curves of ROC between each miRNA and miRNA panel in the validation set.

**Figure 7:**
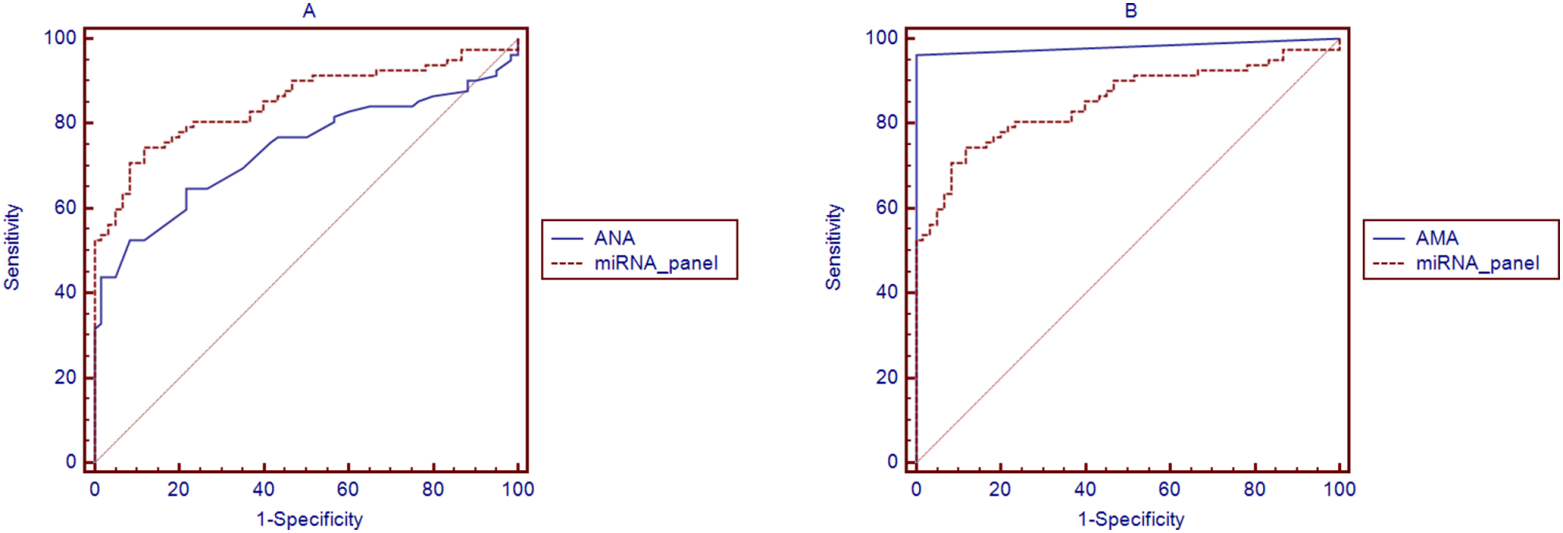
Comparison of the AUC of the miRNA panel with that of ANA and AMA. A: Comparison curves of ROC between ANA and miRNA panel in the validation set; B: Comparison curves of ROC between AMA and miRNA panel in the validation set.

Using the same serum samples, we compared the AUC of the miRNA panel with that of ALP. There was a significant difference between the miRNA panel AUC and ALP AUC (AUC = 0.537, Difference between areas = 0.314, 95% CI = 0.195 to 0.434, *P*<0.001; Fig. 6A). The results indicate that the miRNA panel is a more sensitive and specific biomarker than ALP for PBC. We also compared the AUC of the miRNA panel with that of individual miRNAs (Fig. 6B, Table S4). There was a significant difference between the miRNA panel AUC values and those of individual miRNAs. The results indicate that the miRNA panel has a higher sensitivity and specificity for PBC than has-miR-122-5p, hsa-miR-141-3p, and hsa-miR-26b-5p alone. Moreover, we also compared the miRNA panel AUC with that of ANA and AMA. There was a significant difference between the AUC of the miRNA panel and that of ANA (AUC = 0.739, difference between areas = 0.112, 95% CI = 0.012 to 0.213, *P* = 0.0282; Fig. 7A). However, the sensitivity and specificity of the miRNA panel was less than that of AMA (AUC = 0.982, difference between areas = 0.130, 95% CI = 0.0618 to 0.198, *P* = 0.0002; Fig. 7B).

### Evaluation of the miRNA panel significance in different clinical phases

**Figure 8:**
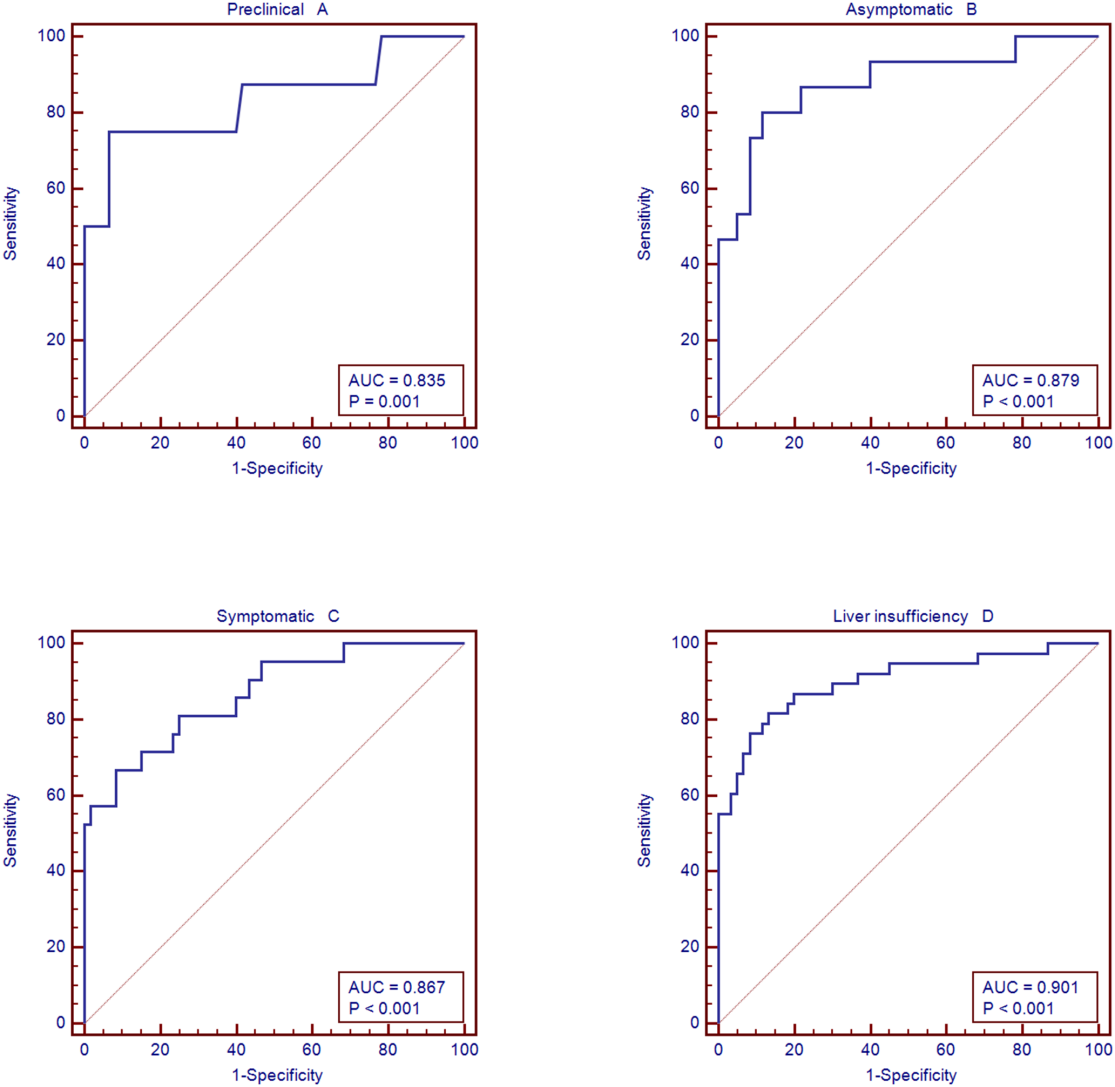
The miRNA panel in different clinical phases in the validation set. A: The corresponding AUCs for patients with preclinical phase, B: The corresponding AUCs for patients with asymptomatic phase, C: The corresponding AUCs for patients with symptomatic phase. D: The corresponding AUCs for patients with liver insufficiency phase.

The diagnostic performance of the miRNA panel in different clinical phases was further evaluated (Fig. 8A–D, Table S5). The corresponding AUCs for patients with different clinical phases (preclinical phase, asymptomatic phase, symptomatic phase, and liver insufficiency phase) were 0.835, 0.879, 0.867, and 0.901, respectively. This indicated that the diagnostic performance of the miRNA panel was independent of the disease status, making it an optimal diagnostic tool.

## Discussion

Since circulating miRNA was found to be stable in serum in 2007, many studies were designed to assess its possible use as a novel and promising biomarker in body fluids. To date, circulating miRNAs have been used as biomarkers in the clinic for cancer detection, non-invasive diagnosis testing, and more [23], [24], [25], [26]. The expression profile of circulating miRNA in serum can be analyzed by qRT-PCR, microarray, and next generation sequencing technolog [27]. Although qRT-PCR has been used as the gold standard to quantify miRNAs, it could only detect limited numbers of miRNAs at once. Microarray, as a high-throughput technology, allows the detection of large numbers of miRNAs, but only known fragments can be detected, and this technology does not detect low versus abundant miRNAs or distinguish miRNAs with single nucleic acid polymorphisms. Compared to these two methods, the next-generation sequencing technology seemed to be more suitable for miRNA profiling. The Roche 454 Genome Sequencer, the Illumina Genome Analyser, and ABI SOLiD System sequencing platforms have become widely available over the past few years.

Recent studies have demonstrated that miRNA expression patterns are disease and tissue specific. miRNAs are abundant in the liver and modulate a diverse spectrum of cellular processes associated with liver injury such as inflammation, apoptosis, and hepatocyte regeneration. Deregulation of miRNA expression may be a key pathogenic factor in many liver diseases, including viral hepatitis, hepatocellular carcinoma (HCC), metabolic and acute liver diseases. miRNA expression profiles are altered in many hepatic diseases compared to that of healthy subject [28], [29], [30], [31], [32]. Hepatocyte apoptosis and necrosis also induce the release of cellular miRNAs directly into the circulation, explaining the superior sensitivity of serum miRNA levels compared to ALT or AST levels in liver damage diagnosis. Bala et al. have shown that miR-122 and miR-155 were predominantly associated with protein aggregates in acetaminophen-induced liver necrosis, whereas in alcoholic liver disease associated inflammatory damag [8], [33]. A number of miRNAs are abundantly expressed in the liver. miR-122 is liver specific. miR-122is estimated to make up 70% of the total hepatic miRNA complement and is expressed at high levels [34]. Therefore, miRNA-122 has been the first to be used in miRNA therapeutic trials since 2008 [35]. Inhibition of miR-122 expression in mice leads to down-regulation of cholesterol- and lipid-metabolizing enzymes [36]. miR-122 is known to regulate metabolic pathways in the liver, including cholesterol biosynthesis [37], [38]. Circulating miR-122 levels have been reported to correlate with liver histological stage, inflammation grades, and ALT activity [34], [35], [36], [38], [39]. miR-141-3p belongs to miR-200 family. Koutsaki et al. [40] showed the aberrant expression of the miR-200 family (miR-200a, miR-200b, miR-200c, miR-141, and miR-429) in ovarian carcinoma and its involvement in ovarian cancer initiation and progression. The miR-200 family members seem to be strongly associated with a pathologic epithelial-mesenchymal transition (EMT) and to have a metastasis suppressive role. Qiu et al. [41] demonstrated that miR-141-3p inhibited human stromal (mesenchymal) stem cell (hMSC) proliferation by G1 cell arrest. miR-141-3p inhibited osteoblast differentiation of hMSC as evidenced by reduced alkaline phosphatase activity, gene expression, and *in vitro* mineralized matrix formation. Yan et al. [42] showed that serum miR-26b-5p may be linked to the toxic effects of perfluorooctanoic acid such as hepatotoxicity, immunotoxicity, and developmental toxicity.

PBC, like most polygenic autoimmune diseases, clearly belongs to the “complex disease” category that is attributable to the combined effects of multiple environmental and behavioral influences and genetic element [43]. All these elements can lead to autoimmune pathology such as PBC. Although many studies demonstrated PBC pathophysiological process, the specific process is still unknown. Recently, some studies have examined the association between PBC and gene expression. MiRNA expression levels have been shown to be significantly different between patients with PBC and healthy control [16], [44], [45]. Qinet et al. [15] analyzed the differential expression profile of microRNA in PBMCs from four PBC patients and four healthy controls using a microRNA array. A total of 17 microRNAs were found to be differentially expressed, 11 microRNAs were upregulated and 6 microRNAs were downregulated in PBC patients. Ninomiya et al. [16] employed Illumina deep sequencing for the initial screening of miRNA expression in 10 PBC, 5 patients with chronic hepatitis B, 5 patients with chronic hepatitis C, and 5 healthy controls. The circulating levels of hsa-miR-505-3p, 197-3p, and 500a-3p were significantly decreased in patients with PBC compared with healthy controls. Thus, more carefully constructed studies are needed to clarify PBC pathogenesis., The analysis of these differentially expressed miRNAs could serve in identifying biomarkers or lead to a better understanding of PBC underlying molecular mechanism [16]. Kerstien et al. [46] also found that a total of 35 independent miRNAs were differentially expressed in PBC and normal liver by histological analysis. The predicted targets of these miRNAs are known to affect cell proliferation, apoptosis, inflammation, oxidative stress, and metabolism.

Compared with other PBS diagnosis studies on circulating miRNAs, our study is unique. First, we screened a large number of serum miRNAs via deep sequencing, which enabled us to better identify potential diagnostic markers. Further, we established a miRNA-panel for PBC diagnosis and revalidated the panel in a large number of serum samples. Moreover, we compared the AUC of the miRNA panel with those of ALP and other miRNAs such asmiRNA-122 and our data indicate that the diagnosis value of the miRNA panel is superior to that of other non-invasive markers in patients with PBC.

In summary, we identified a serum miRNA panel that differentiates patients with PBC from healthy controls with a high degree of accuracy in a large number of participants. Our study demonstrates that this serum miRNA panel has a considerable clinical value for the diagnosis of PBC.

## Supporting Information

Figure S1 **A flowchart outline of study procedures.**
(TIF)

Table S1 **Overview of reads from raw data to cleaned sequences.**
(DOCX)

Table S2 **AUC of ROC curves between PBC and healthy controls in the training set.**
(DOCX)

Table S3 **Logistic regression of miRNAs between patients with PBC and control in training dataset.**
(DOCX)

Table S4 **Comparison of ROC curves between miRNAs panel and miRNAs in the validation set.**
(DOCX)
