# Supplementary figures and images for: Serum MicroRNAs as Potential Biomarkers of Primary Biliary Cirrhosis
Source: PLoS One. 2014 Oct 27;9(10):e111424. doi: 10.1371/journal.pone.0111424 (PMC4210265; doi:10.1371/journal.pone.0111424)

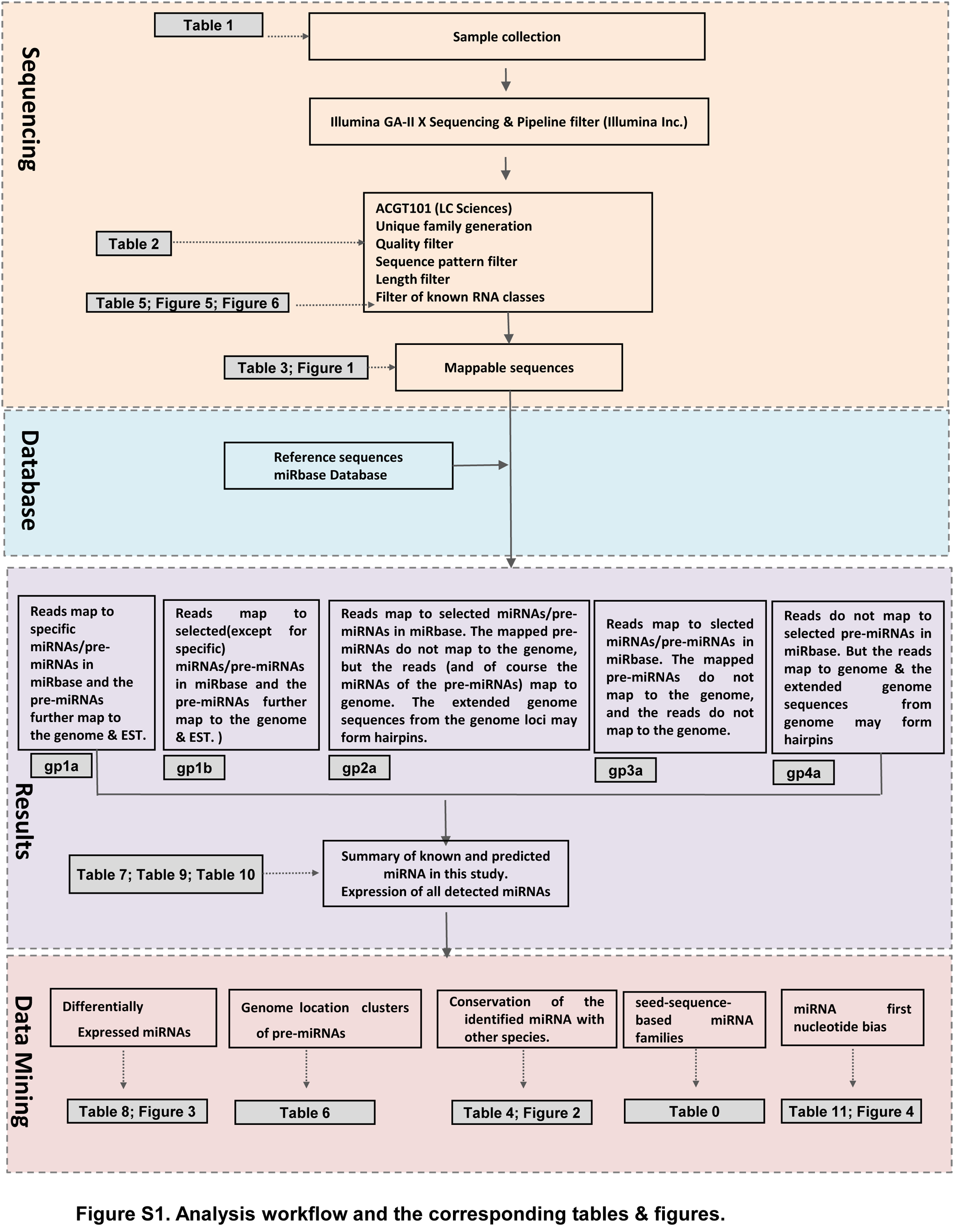

Supplement: Figure S1 — A flowchart outline of study procedures. (TIF) [file pone.0111424.s001.tif]
