# Supplementary material for: Serum MicroRNAs as Potential Biomarkers of Primary Biliary Cirrhosis
Source: PLoS One. 2014 Oct 27;9(10):e111424. doi: 10.1371/journal.pone.0111424 (PMC4210265; doi:10.1371/journal.pone.0111424)
Supplement: Table S1 — Overview of reads from raw data to cleaned sequences. (DOCX) [file pone.0111424.s002.docx]

| Table S1.Overview of reads from raw data to cleaned sequences | | | | | | | | | |
| --- | --- | --- | --- | --- | --- | --- | --- | --- | --- |
| lib | type | Healthy control | | | | PBC | | | |
|  |  | Total | % of Total | uniq | % of uniq | Total | % of Total | uniq | % of uniq |
| Raw reads | NA | 8580434 | 100 | 1181248 | 100 | 9371001 | 100 | 944362 | 100 |
| 3ADT&length filter | Sequence type | 1070277 | 12.47 | 389134 | 30.01 | 1309765 | 13.98 | 327392 | 33.4 |
| Junk reads | Sequence type | 4338 | 0.05 | 2221 | 0.19 | 3902 | 0.04 | 1792 | 0.19 |
| Rfam | RNA class | 658730 | 7.68 | 76634 | 6.31 | 524967 | 5.6 | 69142 | 7.23 |
| mRNA | RNA class | 335068 | 3.91 | 67548 | 5.56 | 333047 | 3.55 | 68354 | 7.15 |
| Repeats | RNA class | 158543 | 1.85 | 9839 | 0.81 | 105632 | 1.13 | 9828 | 1.03 |
| rRNA | RNA class | 265420 | 3.09 | 39640 | 0.45 | 207976 | 2.22 | 35413 | 0.38 |
| tRNA | RNA class | 235763 | 2.75 | 16877 | 0.18 | 177572 | 1.89 | 14467 | 0.15 |
| snoRNA | RNA class | 28791 | 0.34 | 4711 | 0.05 | 20524 | 0.22 | 4550 | 0.05 |
| snRNA | RNA class | 29497 | 0.34 | 3383 | 0.04 | 25098 | 0.27 | 3244 | 0.03 |
| other Rfam RNA | RNA class | 99259 | 1.16 | 12023 | 0.14 | 93797 | 1 | 11468 | 0.12 |
| Clean reads | Sequence type | 6628837 | 77.26 | 659447 | 54.26 | 7319212 | 78.1 | 482263 | 50.29 |
