# Supplementary material for: Serum MicroRNAs as Potential Biomarkers of Primary Biliary Cirrhosis
Source: PLoS One. 2014 Oct 27;9(10):e111424. doi: 10.1371/journal.pone.0111424 (PMC4210265; doi:10.1371/journal.pone.0111424)
Supplement: Table S2 — AUC of ROC curves between PBC and healthy controls in the training set. (DOCX) [file pone.0111424.s003.docx]

| Table S2 AUC of ROC curves between PBC and healthy controls in training set | | | | | | |
| --- | --- | --- | --- | --- | --- | --- |
| Variable | AUC | 95% CI | Sencitivity | Specificity | z statistic | *p* |
| hsa-miR-122-5p | 0.788 | 0.723 to 0.854 | 72.5 | 81.1 | 8.6 | <0.0001 |
| hsa-miR-34a-5p | 0.662 | 0.583 to 0.741 | 66.7 | 68.9 | 4.018 | 0.0001 |
| hsa-miR-141-3p | 0.647 | 0.569 to 0.726 | 45.1 | 86.7 | 3.689 | 0.0002 |
| hsa-miR-26b-5p | 0.791 | 0.723 to 0.859 | 76.5 | 86.1 | 8.399 | <0.0001 |
| hsa-miR-27b-3p | 0.571 | 0.491 to 0.652 | 30.4 | 86.7 | 1.731 | 0.0834 |
