# Supplementary material for: Serum MicroRNAs as Potential Biomarkers of Primary Biliary Cirrhosis
Source: PLoS One. 2014 Oct 27;9(10):e111424. doi: 10.1371/journal.pone.0111424 (PMC4210265; doi:10.1371/journal.pone.0111424)
Supplement: Table S3 — Logistic regression of miRNAs between patients with PBC and control in training dataset. (DOCX) [file pone.0111424.s004.docx]

| Table S3 Logistic regression of miRNAs between PBC and control in training dataset | | | | | |
| --- | --- | --- | --- | --- | --- |
| Variable | Coefficient | Std. Error | Odds ratio | 95% CI | P |
| has-miR-122-5p | -0.74034 | 0.12312 | 0.477 | 0.3747 to 0.6071 | <0.0001 |
| has-miR-141-3p | -0.3616 | 0.090329 | 0.6966 | 0.5415 to 0.8961 | 0.0049 |
| has-miR-26b-5p | 0.65338 | 0.12346 | 1.922 | 1.5089 to 2.4482 | <0.0001 |
| Constant | 10.2834 |  |  |  |  |
| Enter variable if P< 0.05 ,remove variable if P> 0.1;Overall model fit:Null model -2 Log Likelihood =265.418 ;Full model -2 Log Likelihood= 167.272 ;*x*^2^ =98.146 ,*P* < 0.0001.  logitP=10.2834 - 0.74034miR122 - 0.3616miR141 + 0.65338miR26b AUC=0.876,95%CI 0.821 to 0.919 | | | | | |
|  |  |  |  |  |  |
|  |  |  |  |  |  |
|  |  |  |  |  |  |
|  |  |  |  |  |  |
|  |  |  |  |  |  |
|  |  |  |  |  |  |
