# Supplementary material for: Serum MicroRNAs as Potential Biomarkers of Primary Biliary Cirrhosis
Source: PLoS One. 2014 Oct 27;9(10):e111424. doi: 10.1371/journal.pone.0111424 (PMC4210265; doi:10.1371/journal.pone.0111424)
Supplement: Table S4 — Comparison of ROC curves between miRNAs panel and miRNAs in the validation set. (DOCX) [file pone.0111424.s005.docx]

| Table S4 Comparison of ROC curves between miRNAs panel and miRNAs in validation set | | | | | |
| --- | --- | --- | --- | --- | --- |
| Variable | AUC | Difference between areas | 95% CI | z statistic | *p* |
| has-miR-122-5p^a^ | 0.778 | 0.127 | 0.0710 to 0.183 | 4.433 | 0.0001 |
| has-miR-141-3p^b^ | 0.659 | 0.246 | 0.164 to 0.329 | 5.848 | <0.0001 |
| has-miR-26b-5p^c^ | 0.82 | 0.0847 | 0.0139 to 0.155 | 2.344 | 0.0191 |
| miRNA-panel | 0.905 |  |  |  |  |
| Pairwise comparison, ^a^miRNA-panel & has-miR-122_5p; ^b^miRNA-panel & has-miR-141-3p; ^c^miRNA_panel & has-miR-26b-5p. | | | | | |
